# Neural Network Based Mental Depression Identification and Sentiments Classification Technique From Speech Signals: A COVID-19 Focused Pandemic Study

**DOI:** 10.3389/fpubh.2021.781827

**Published:** 2021-12-06

**Authors:** Syed Thouheed Ahmed, Dollar Konjengbam Singh, Syed Muzamil Basha, Emad Abouel Nasr, Ali K. Kamrani, Mohamed K. Aboudaif

**Affiliations:** ^1^School of Computing and Information Technology, REVA University, Bengaluru, India; ^2^ICT for Internet and Multimedia, University of Padua, Padua, Italy; ^3^School of Computer Science and Engineering, REVA University, Bengaluru, India; ^4^Industrial Engineering Department, College of Engineering, King Saud University, Riyadh, Saudi Arabia; ^5^Industrial Engineering Department, College of Engineering, University of Houston, Houston, TX, United States

**Keywords:** sentiment extraction, speech signal processing, COVID-19, mental depression, neural network

## Abstract

COVID-19 (SARS-CoV-2) was declared as a global pandemic by the World Health Organization (WHO) in February 2020. This led to previously unforeseen measures that aimed to curb its spread, such as the lockdown of cities, districts, and international travel. Various researchers and institutions have focused on multidimensional opportunities and solutions in encountering the COVID-19 pandemic. This study focuses on mental health and sentiment validations caused by the global lockdowns across the countries, resulting in a mental disability among individuals. This paper discusses a technique for identifying the mental state of an individual by sentiment analysis of feelings such as anxiety, depression, and loneliness caused by isolation and pauses to the normal chains of operations in daily life. The research uses a Neural Network (NN) to resolve and extract patterns and validate threshold trained datasets for decision making. This technique was used to validate 2,173 global speech samples, and the resulting accuracy of mental state and sentiments are identified with 93.5% accuracy in classifying the behavioral patterns of patients suffering from COVID-19 and pandemic-influenced depression.

## Introduction

The world is at present facing an uncertain time due to the global pandemic caused by Severe Acute Respiratory Syndrome Coronavirus 2 (SARS-CoV-2), also known as COVID-19. The pandemic has forced nations to exercise lockdown as a preventive measure to slow the spread of the virus. The pandemic has resulted in economic failure and disruptions in the supply chain all over the world. There was a race for a vaccine among modern drug and research organizations. The pandemic has caused major adverse effects such as mental depression, isolation, anxiety, and loneliness besides respiratory disorders and aligned symptoms. Depression and other mental health issues have been caused by lockdown and restrictions in travel and work, with a new normal social life now conducted via technological platforms.

The pandemic has bought a sense of maturity and adverse implications concerning psychosocial behaviors and mental health implications such as depression, anxiety, and loneliness. In this research, a systematic evaluation was conducted on the behavior of users based on speech signals, which were recorded using a machine learning technique to extract keywords and classify data using sentimental analysis techniques (1). The research in this article also focuses on identifying the user's mental state via the speech signals recorded over technological platforms used for virtual meetings and other gatherings (2). The research aims to provide schematic evaluation and validation approaches and classify patients based on medical conditions.

## Literature Survey

The global pandemic situation under COVID-19 has left traces of various adverse effects on people, which are the result of isolation, lockdown, mental health destabilization, and much more. The authors Singh et al. (3) and Naik et al. (4) have discussed the impact caused by COVID-19 and lockdown on the younger generation, focussing on children's behavior and reactions to the new normal. The study shows the overall implications of isolation on children and adolescents. Pfefferbaum and North (5) have discussed the impact and relationship of mental stress caused due to the global pandemic situation, with detailed insights into public health emergencies and the influence of the pandemic on looming health conditions. Furthermore, a discussion on the challenges faced by health care workers (HCW) and their state of mental stress is documented by Spoorthy et al. (6). The HCWs are frontline attributes and hence require assistance in evaluating and validating mental health via the main mode of communication now used, i.e., speech signals through digital media platforms and applications and a similar discussion is highlighted in other studies (7, 8).

Some studies are focused on the terms of technological solutions for the mental distress caused by the pandemic. These solutions have outlined the use of a telemedicine approach for reaching the maximum and remote population of a developing country like India. A study by Ahmed et al. (9) discussed Multidimensional Optimal Medical Dataset processing under a telemedicine channel. These MooM datasets include a signal processing unit for a standardized approach and can be used for intimated processing in the proposed study, with a supported algorithm from (10). The method of detecting and validating speech signals is also proposed in this article, based on the influence of telemedicine approaches with a numerical clustering validation by (11) and (12).

The latest findings in the survey are recorded with real-time datasets as discussed in (4). This approach aims to validate treatment and handling, focusing on pandemic control and coordination. The prediction and modeling of the pandemic are discussed by Iwendi et al. (13) and Ngabod et al. (14), who propose a technique for classifying pandemic growth in smart cities. Under the process processing state, this dedicated networking model can be utilized, as discussed by Ahmed et al., under a dynamic user cluster grouping approach (15–17). These developments have provided a reliable solution for handling pandemic data using text mining and decision support. The classification of Covid-19 studies and surveys are reported and validated by (18, 19).

## Methodology

**Figure 1:**
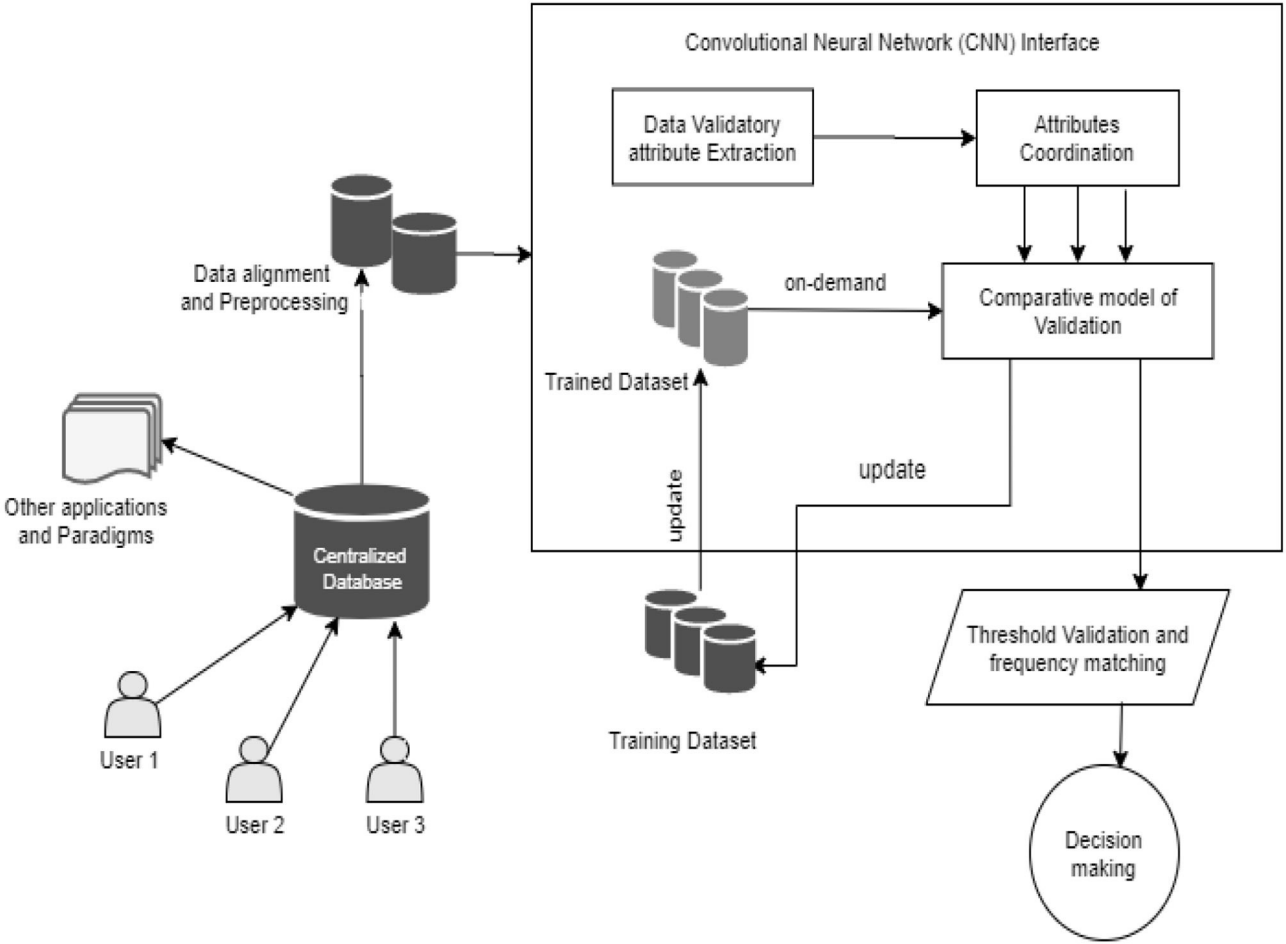
Architectural diagram of the proposed technique toward decision making in speech signals.

The proposed methodology aims to focus on the detection and validation of speech signals via a depression and mental disorder identification based on speech signal processing using a neural network (NN). The process is defined using mass datasets from 2,173 speech samples, as discussed in the architecture model in Figure 1. The agenda of the proposed technique is to restore a correlation with trained datasets in extracting and evaluating the samples of speech and classifying on demand. These speech signals are interdependent and have a higher order of distinction in recovering and validating the sample of COVID-19 patients' mental stability and sentiments (20).

The processing datasets are computed in a centralized database with user-to-user interface coordination, thereby generating a pool of databases consisting of raw and unprocessed data from the users. The process is initiated with data alignment and pre-processing techniques, as discussed in the mathematical modeling of the proposed technique. The process is designed with a trained database of the speech signals with a heap address of thresholds relating to global attributes such as country, location, gender, age, and professional practice.

**Figure 2:**
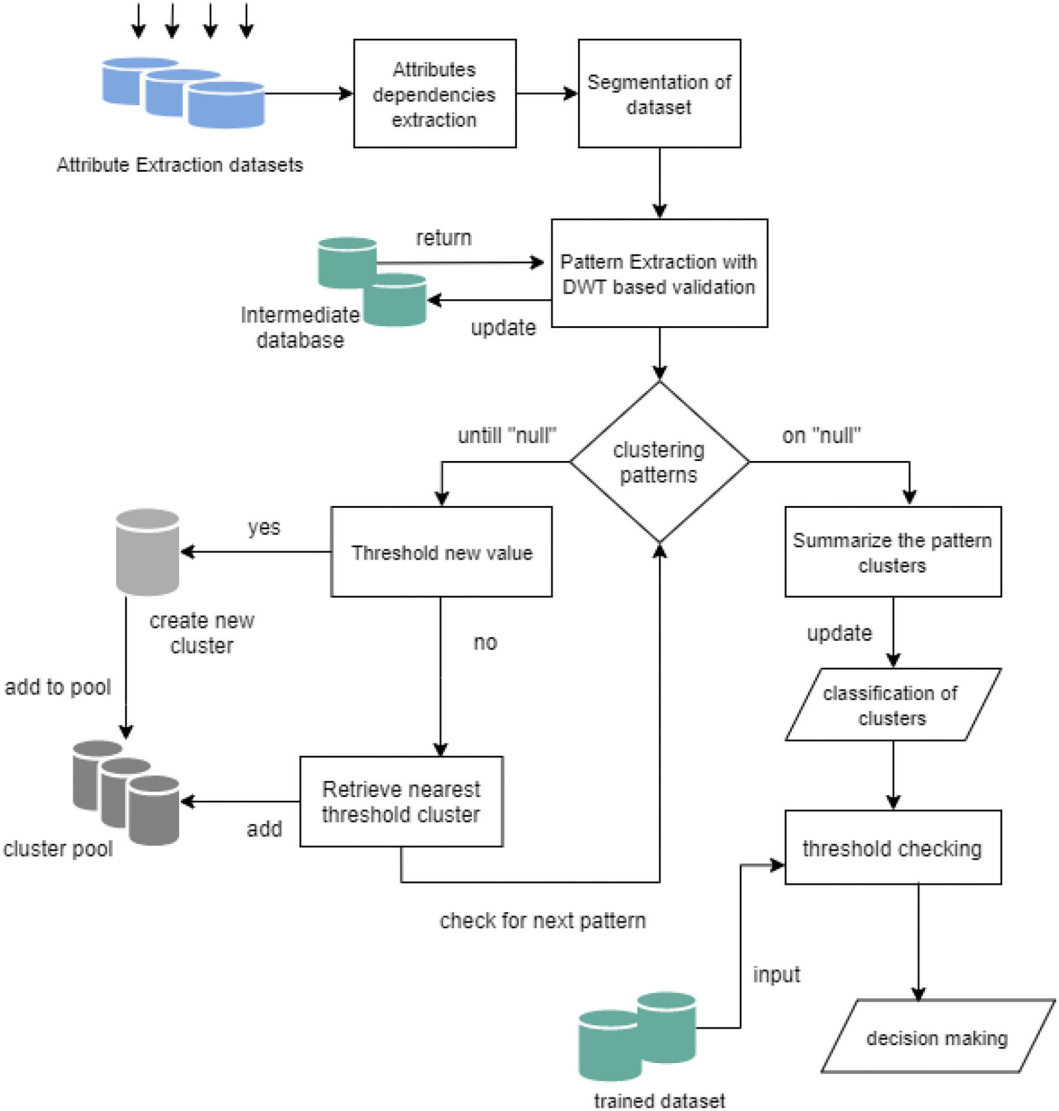
Comparative model for validating speech signals in distress detection.

**Figure 3:**
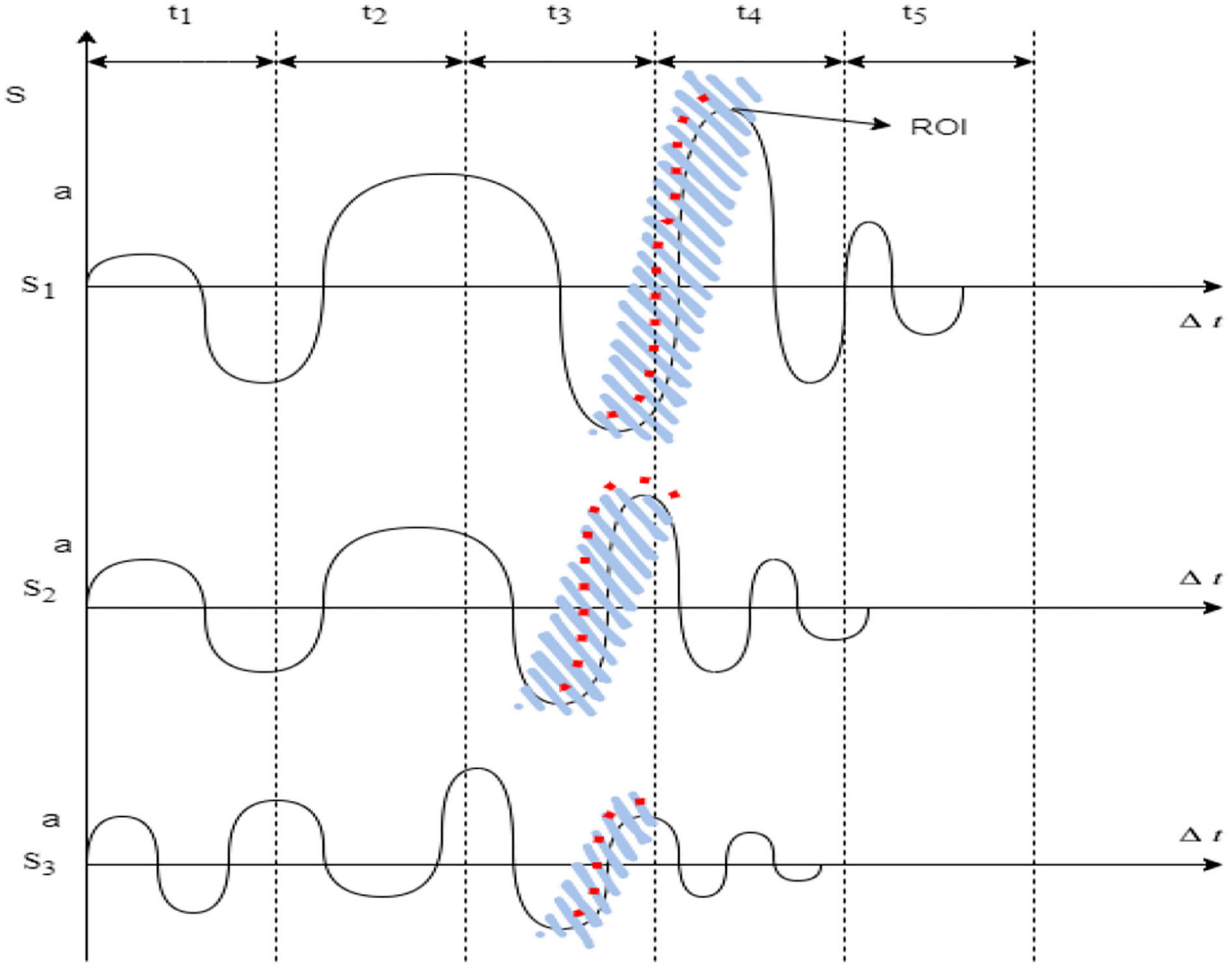
ROI on floating speech samples of multi-users.

The trained datasets provide the threshold process for the extracted attributes of the user input signals. The process is designed with a comparative validation model to assure the process execution, as demonstrated in Figure 2. The comparative model evaluates the detailed execution process, such as the pattern extraction and clustering of signal samples in the form of JPEG intermediate files and a dedicated intermediate database, generating a cluster pool for segregation of samples based on ROI as demonstrated in Figure 3. This results in a threshold value comparison and thus provides a single decision and classification of the user's mental condition.

## Mathematical Representation

The computation of the speech signals and detection of mental stress is achieved under the processed instruction architecture, as demonstrated in Figure 1. The process aims to validate the signals into coordination datasets with a synchronization approach of proving learning and pooling clusters of similar patterns, as demonstrated in Figure 3. The mathematical approach is discussed in this section.

### Attribute Extraction and Dependencies Validation

Consider a dataset *(D)* with a raw calibrated ecosystem of attributes *(A)* where each of attributes *A*=*{A*_1_*,A*_2_*,A*_3_*,……,A*_*n*_*}* such that each attribute *(A*_*i*_*)* resembles the paradigm of operation, as in Equation (1).


(1) $$\begin{array}{c} A_{i}=\int_{0}^{\infty }\frac{\partial (A_{i})}{\partial (D)} \end{array}$$

Where, each of the *i*^*th*^ attributes, has a correlated paradigm of operation and process extraction. Thus, the extracted attributes *(A*_*e*_*)* are as shown in Equation (2).


(2) $$\begin{array}{c} A_{e}=\int_{0}^{\infty }(\Delta D_{z}.{\sum^{\;}}_{i=0}^{n}\frac{\partial A_{i}}{\partial D}).\Delta T \end{array}$$

Where the extracted attribute *(A*_*e*_*)* is processed over the raw attributes set, in extracting the most relevant threshold attributes such as the peak frequency of a word or a repeated phrase of a sentence with a dilution of Δ*D*_*z*_ and mapping with Δ*T* as a threshold paradigm in validating all processing attributes *(A*_*e*_*)* in the speech signal.

### Segmentation of Samples

Samples are primarily divided into extracted attributes *(A*_*e*_*)* sets, such that each of the attribute Region of Interest (ROI) is highlighted and marked in the entire speech signal, as shown in Figure 3.

Consider the segmentation *(S)* of the overall input signal (speech signal) with a highlighted extracted attribute *(A*_*e*_*)*. On consideration, each attribute in the signal has an occupancy time *(*Δ*t)* in operating, and thus, a reflective ratio of division is processed based on CNN's evaluation paradigms.

The signal *(S)* of an independent sample *(S*_*i*_*)* tends to occur in ROI in an independent location of the time matrix *(*Δ*t)*. Hence, the segmentation of signal *(S)* is as shown in Equation (4).


(3) $$S=\frac{2\pi }{\Delta R}\int_{0}^{\infty }\left[\int_{n}^{i}\left(\frac{\Delta S_{i}}{\Delta t}\right)\right].\frac{\Delta t}{\Delta s}$$

(4) $$\begin{array}{c} ∴S=\frac{2\pi }{\Delta R.\Delta S}\left(\int_{0}^{\infty }(\frac{\partial S_{i}}{\partial t}).\frac{\partial (A_{e})}{\partial t}\right).\Delta t \end{array}$$

Where each signal strength is measured in Δ*R* with a signal time Δ*t*, for all regional attributes extraction; hence, for segmentation to be processed completely, the schematics of each attribute signal strength *(*Δ*R)* is then computed with an exhausted peak of ROI from the signal as shown in Figure 3.

### Pattern Extraction and Schema Alignment of Datasets

The process of pattern extraction is calibrated with the internally divided segments of datasets. These datasets are processed with a frequency *(f)* such that the internal segments *(S)*= *{S*_1_*,S*_2_*,S*_3_*,…….,S*_*n*_*}* has calibrated frequencies *(f)*=*{f*_1_*,f2,f*_3_*,……,f*_*n*_*}*. Thus, the inter-correlated frequencies can be defined and associated as *(S*_*f*_*)*=*{S*_*f*1_*,S*_*f*2_*,S*_*f*3_*,……,S*_*fn*_*}*.

The process of pattern with speech signals is internally correlated to the amplitude of the signal *(amp)*. Where it is represented as *f*_*amp*_ =*{f*_*amp*1_*,f*_*amp*2_*,f*_*amp*3_*,…..,f*_*ampn*_*}*. The amplitude of each frequency feature can be represented and extracted as shown in Equation (5).


(5) $$\begin{array}{l} S_{i}\left(n\right)=\;\int \left\{\sum_{k=0}^{n}\left[\alpha_{i}\left(k\right).amp\left(f\right)\right]\right\} \end{array}$$

Where each signal pattern *(S*_*n*_*)* represents the overall coordination in speech signals, and the ‘' represents band filters of the speech signal with a coefficient of amplitude and frequency. On extraction of patterns from those correlated in Equation (5), the frequency patterns can be sorted by independent bandwidth as shown in Equation (6).


(6) $$\begin{array}{c} P_{i}=\frac{2\pi }{\Delta R.\Delta S}\{(\log(G(f)).\int_{0}^{\infty }\frac{\partial (S_{i}(n))}{\partial t}{)}^{2}\} \end{array}$$

(7) $$\begin{array}{c} P_{i}=\frac{2\pi }{\Delta R.\Delta S}\{(\log{\left(G(f_{amp})\right)}^{2}.\int_{0}^{\infty }\frac{\partial^{2}(S_{i}(n))}{\partial t^{2}})\} \end{array}$$

The ‘*P*_*i*_' on Equation 7 is the pattern of repeated learning from the CNN framework. The internal arrangements can be represented as the frequency *(f)* under the operation of amplitude, (mode) is represented as *f*_*amp*_, further graded into the Gaussian constant *(G)*. The process in Equation (7) is then concluded, as shown in Equation (8).


(8) $$\begin{array}{l} P_{i}=\frac{2\pi }{\Delta R.\Delta S}\{(2log(G(f_{amp})).\\ \;\int_{0}^{\infty }\frac{\partial^{2}({\sum^{\;}}_{j=0}^{n}{\sum^{\;}}_{k=0}^{n}\alpha_{i}(j).amp(f_{k}))}{\partial t^{2}})\} \end{array}$$

(9) $$\begin{array}{l} P_{i}=\frac{4\pi }{\Delta R.\Delta S}\{(\log G(f_{amp}).\\ \;\int_{0}^{\infty }\frac{\partial^{2}({\sum^{\;}}_{j=0}^{n}\langle {\sum^{\;}}_{k=0}^{n}\alpha_{i}(j).amp(f_{k})\rangle )}{\partial t^{2}})\} \end{array}$$

(10) $$\begin{array}{l} P_{i}=\frac{4\pi .logG(f_{amp})}{\Delta R.\Delta S}.\\ \;\{(\int_{0}^{\infty }\frac{\partial^{2}({\sum^{\;}}_{j=0}^{n}\{{\sum^{\;}}_{k=0}^{n}\langle \alpha_{i}(j).amp(f_{k})\rangle \})}{\partial t^{2}})\} \end{array}$$

Thus, Equation (10) represents the coordinates of the pattern extracted and validated for the pattern with respect to a segment *(S*_*i*_*)*. Thus, on summarisation, the representation can be as *P* = *{P*_1_*,P*_2_*,P*_3_*,….,P*_*n*_*}* co-related to coordination of segment as *S*_*p*_=*{S*_*p*1_*,S*_*p*2_*,S*_*p*3_*,……,S*_*pn*_*}*, where ‘*n'* is the last segment of given input signal.

### Clustering and Classification of Datasets

**Figure 4:**
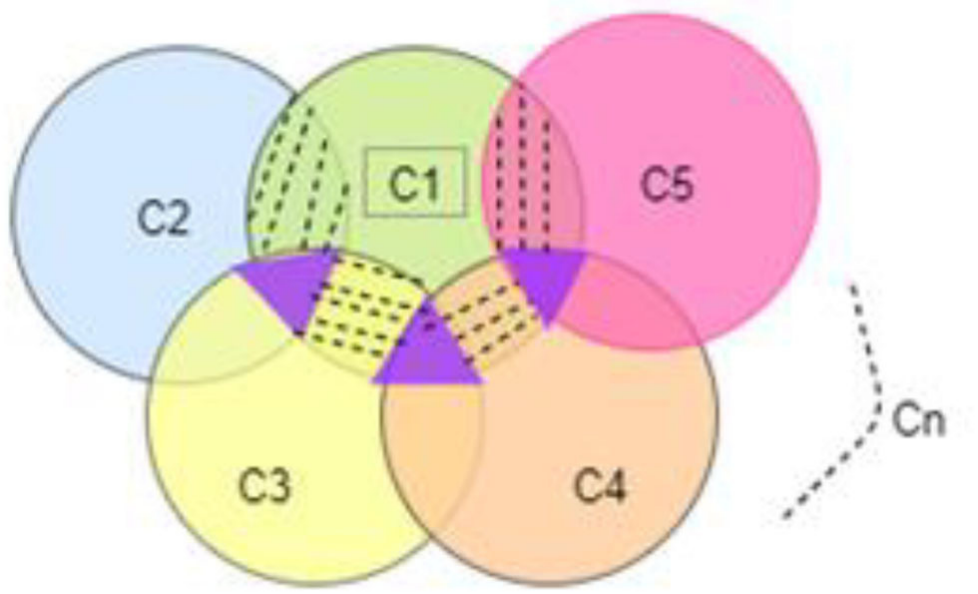
Cluster representation of extracted patterns.

Equation (10) retrieves the pattern of individual segments, and thus the coefficient of such segments are summarized and represented in *S*_*p*_=*{S*_*p*1_*,S*_*p*2_*,S*_*p*3_*,……,S*_*pn*_*}*. Hence the clustering is shown in Figure 4.

The cluster *(C)* is retained from a group of values and its corresponding coefficients for the value re-compensation. The clusters are internally evaluated with the focus of associating.


(11) $$\begin{array}{c} C_{i}=\int_{0}^{\infty }\left\{{\sum^{\;}}_{i=0}^{n}[{\sum^{\;}}_{j=i}^{n}(\frac{\partial {\left(P_{i}\right)}_{j}}{\partial t}).\Delta T_{i}]\right\} \end{array}$$

Where each cluster *(C*_*i*_*)* is validated with a corresponding pattern coefficient and a threshold value *(*Δ*T)*. The internal Threshold value *(*Δ*T)* is validated and evaluated. In summary, the clusters *(C)* = *{C*_1_*,C*_2_*,C*_3_*,…..,C*_*n*_*}*. These clusters have an association of common patterns, for example, represented as *{(C*_*i*_∩ *C*_*j*_*)* ∩ *C*_*k*_*}*, and these associations are subjected to attribute validation, as shown in Figure 4.

### Threshold Validation and Decision Making

The clusters and classification of speech signals using clusters are validated and approved for processing into decision making. The decision-making approach is termed with a threshold value consultation, i.e., the overall technique extracts the validated pattern coefficient and thereby synchronizes it with a relatively more and likely approach of matching and schema validation. The proposed approach typically validates the decision of signal segmentation using the threshold value toward segregating the dataset of speech signals based on emotions. These emotion-based evaluations are rather computational, and hence a most likely decision is processed.

## Results and Discussions

The proposed technique has successfully retrieved the signal attributes and the prediction ratio for evaluation. The input signals from the users via a remote connecting platform are uploaded to a centralized database in a cloud computing ecosystem using AWS-sponsored services. The datasets are processed and validated according to a multidimensional approach.

**Figure 5:**
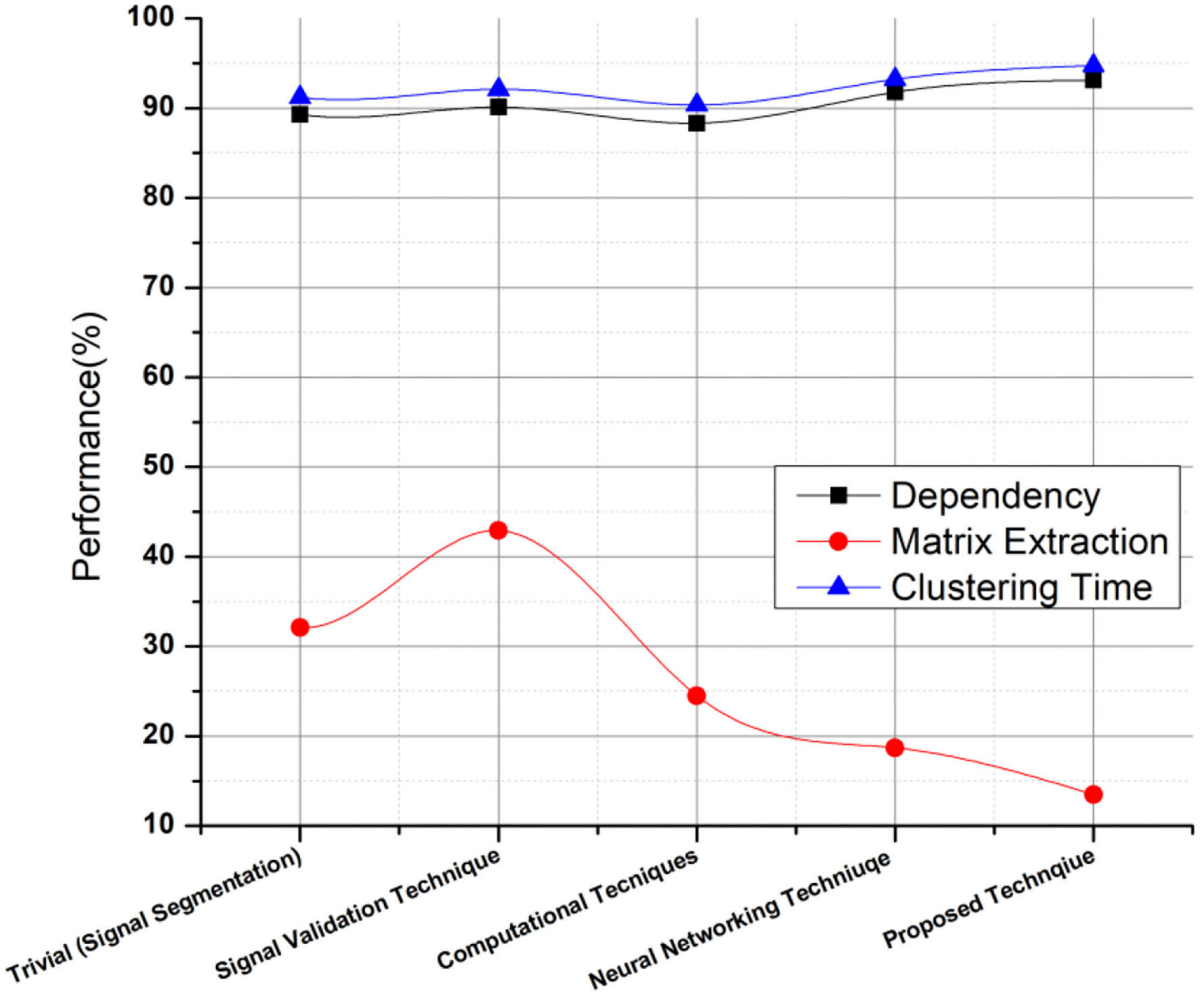
Performance computation of proposed technique on independent parameters.

**Figure 6:**
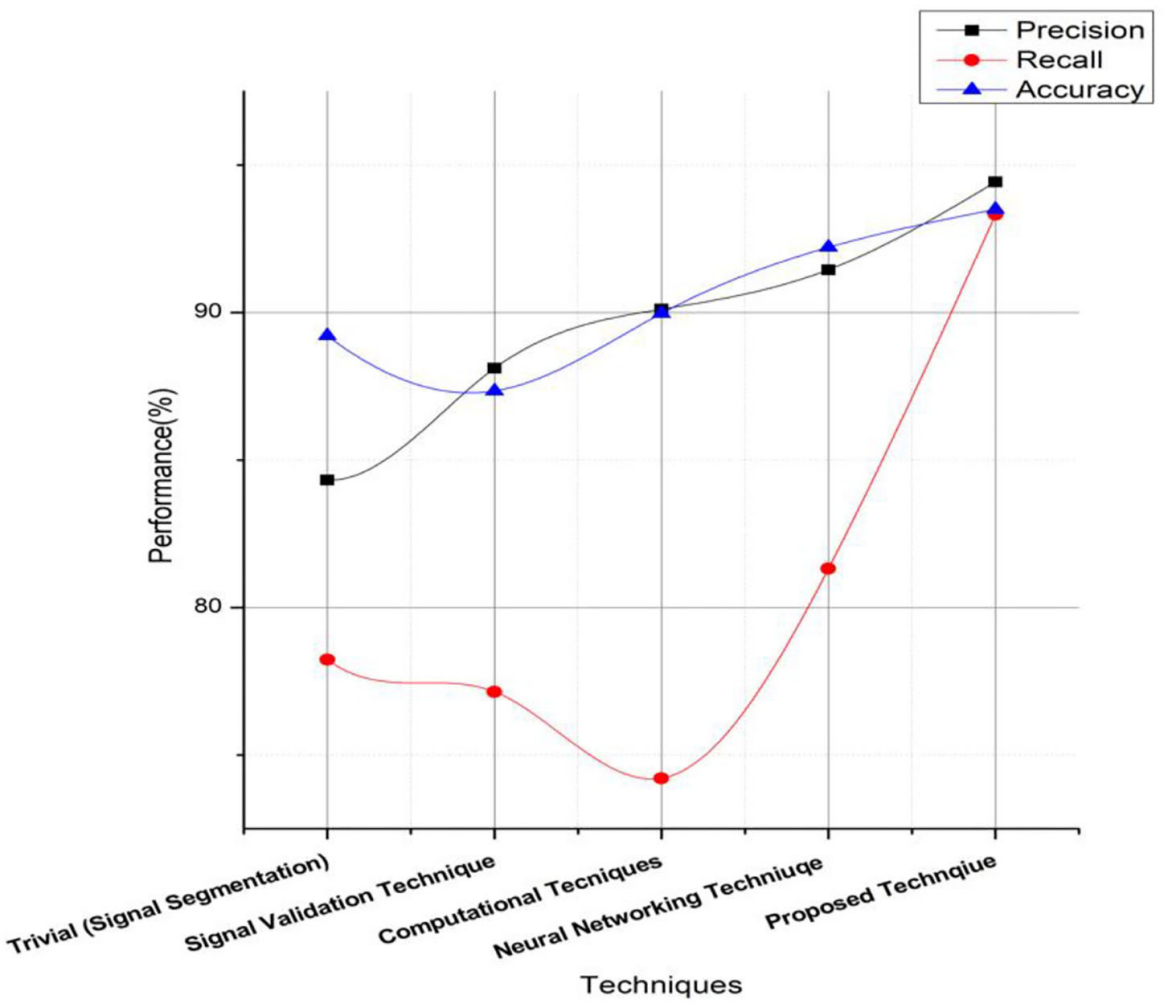
Outcome evaluation of proposed technique.

The variation of predicting the sentiments is based on the information designed and developed via clustering datasets. The prediction ratio is summarized in Figures 5, 6, respectively, with a comparative evaluation from previous systems. Table 1 shows the parameters related to the mental stress and paradigms to provide decision support. The table highlights the evaluation parameters such as the occurrence delay of a keyword in clustering, as shown in Equation (11). The supported approach thus classifies the pattern of these keyword occurrence sequences for decision making.

**Table 1:** Decision support and evaluation parameters.

**Patient relevance (sentiments)**	**Cluster range**	**Occurrence difference (Avg. sec)**	**Pattern differences**	**Decision accuracy (%)**
COVID−19 (positive)	Average	3.211	0.327	97.23
Post COVID−19 (positive)	High	1.432	0.129	94.92
Loneliness	High	1.328	0.091	96.91
Anxiety	Average	2.114	0.181	92.17
Depression	High	0.994	0.021	97.28
Normal (Non-COVID19)	Low	5.251	0.448	95.39

The results of data/signal processing and decision-making are shown in Table 2. The results show promising outcomes in proving a precision of 90% and higher in various users across the language and location. The results of processing a single sample are included in Table 3. The processing signal magnitude and the power spectrum computation demonstrate a higher order of signal clarity in analysis and validation.

**Table 2:** Performance matrix for speech signal in mental distress validation.

**Sample (Age group) (yrs)**	**Approach**	**Precision (%)**	**Recall (%)**	**Detection-score (%)**	**Accuracy (%)**
5–9	Unclassified	92.43	89.23	89.3	90
10–15	Unclassified	95.72	93.22	93.08	95
16–25	Classified	97.12	97.48	98.1	98.48
26–35	Classified	97.78	97.53	98.9	98.97
36–55	Classified	98.3	97.12	99.12	99.23
56–60	Classified	95.33	91.23	97.12	94.19
61–75	Unclassified	96.12	89.23	91.07	88.32
76–100	Unclassified	92.31	88.7	87.2	81.2

**Table 3:** Signal processing and analysis appendix.

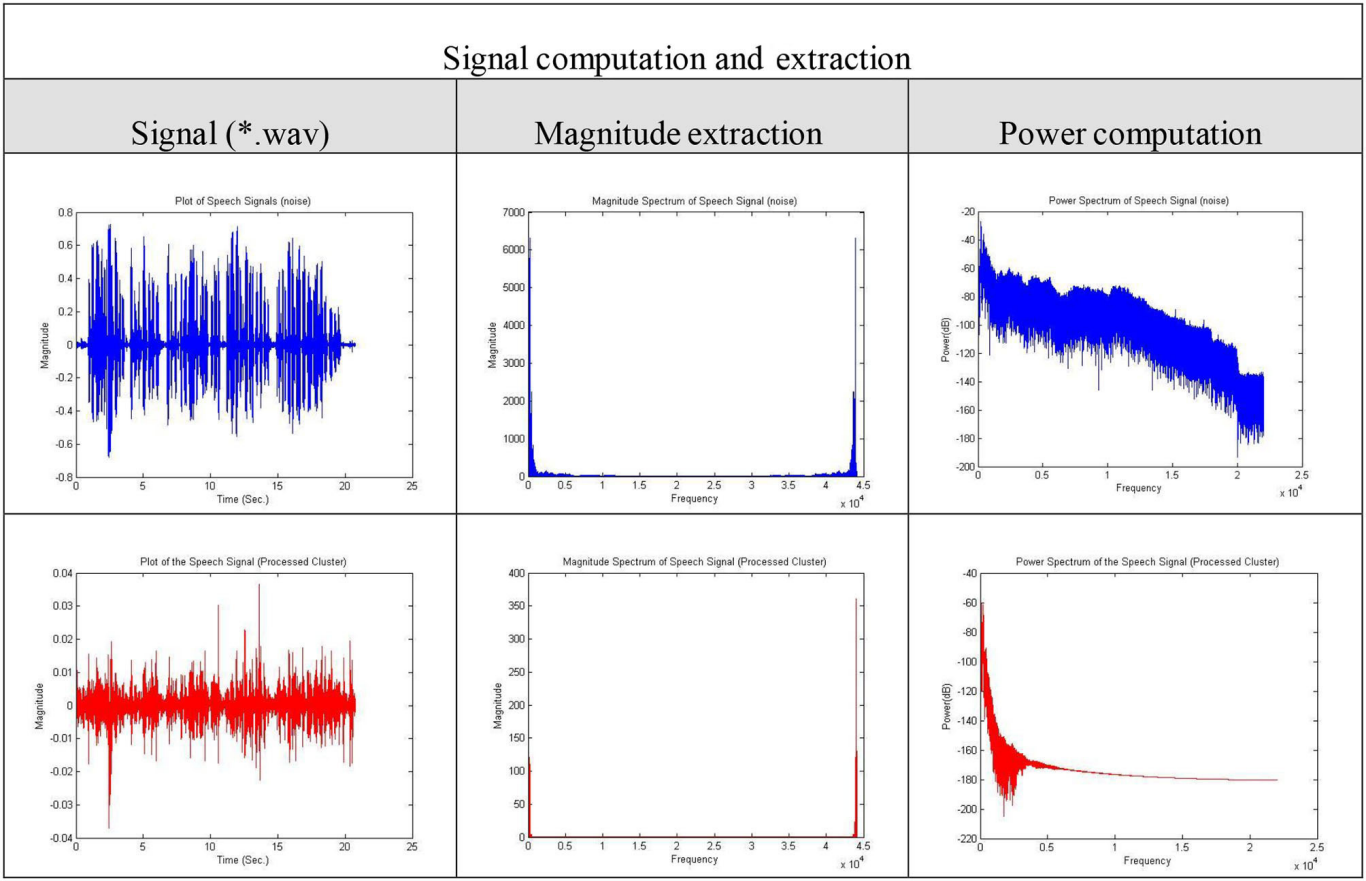		

## Conclusion

The technique proposed in the present study uses neural networking terminology to learn and develop a pool of clusters and patterns to provide a systematic and reliable decision to categorize speech signals. The processing system is based on open database processing to validate the mental health conditions of users during the ongoing isolation and lockdowns caused by the COVID-19 pandemic. The results show a promising outcome with a precision of 90% and higher accuracy across various users. The approach has a projected accuracy of 93.5% under the open validation platform on a computational evaluation. The proposed technique could be included in classifying and categorizing patients' behavior in future, with supervised approaches to keyword extraction and classification in dynamic signals.

## Data Availability Statement

The original contributions presented in the study are included in the article/supplementary material, further inquiries can be directed to the corresponding authors.

## Author Contributions

Conceptualization and writing: SA, DS, SB, EA, and MA. Methodology: SA, DS, SB, AK, and MA. Investigation and programming: SA and DS. Resources: SB, EA, MA, and AK. Review: EA and AK. All authors contributed to the article and approved the submitted version.

## Funding

The authors extend their appreciation to King Saud University for funding this work through Researchers Supporting Project number (RSP-2021/164), King Saud University, Riyadh, Saudi Arabia.

## Conflict of Interest

The authors declare that the research was conducted in the absence of any commercial or financial relationships that could be construed as a potential conflict of interest.

## Publisher's Note

All claims expressed in this article are solely those of the authors and do not necessarily represent those of their affiliated organizations, or those of the publisher, the editors and the reviewers. Any product that may be evaluated in this article, or claim that may be made by its manufacturer, is not guaranteed or endorsed by the publisher.
